# Cultivation of elusive microbes unearthed exciting biology

**DOI:** 10.1038/s41467-020-20393-9

**Published:** 2021-01-04

**Authors:** Muriel C. F. van Teeseling, Christian Jogler

**Affiliations:** 1 Department of Biology, University of Marburg, Marburg, Germany; 2 Institute of Microbiology, Department of Microbial Interactions, Friedrich Schiller University Jena, Jena, Germany

**Keywords:** Cell biology, Cellular microbiology, Bacteriology, Environmental microbiology

## Abstract

Many newly-discovered microbial phyla have been studied solely by cultivation-independent techniques such as metagenomics. Much of their biology thus remains elusive, because the organisms have not yet been isolated and grown in the lab. Katayama et al. lift the curtain on some intriguing biology by cultivating and studying bacteria from the elusive OP9 phylum (*Atribacterota*).

With the advent of molecular techniques, it became evident that there are far more microbes than scientists have managed to cultivate in the laboratory^1^. A dogma emerged that >99% of this ‘microbial dark matter’ is impossible to cultivate and thus can only be studied with cultivation-independent omics techniques. Such omics approaches have been extremely successful in expanding the known microbial tree of life, showing that the majority of microbial diversity is not represented by model organisms^2^. This diversity is not limited to the rare biosphere but includes abundant organisms, some of which are important for human health and wellbeing.

However, although omics techniques provide a powerful starting point, uncovering the microbiological treasure trove requires cultivation in the laboratory^3^. The dogma of the ‘impossible microbes’ has luckily been challenged by successful cultivation of microorganisms such as anammox bacteria^4^, an Asgard archaeon^5^, a member of the candidate phyla radiation^6^ and extremely diverse Planctomycetes^7,8^. Obtaining these cultures was labor-intensive and required patience, but their study has altered our understanding of microbial physiology, cell biology, and evolution. Now, Katayama et al.^9^ report the cultivation of *Atribacter laminatus*, thus adding another thought-provoking organism to the microbial zoo of curiosities.

## *Atribacter laminatus*: the latest cultivation success

Using culture-independent techniques, the bacterial candidate phylum OP9 was previously discovered in hot spring sediments in Yellowstone National Park^1^, and later found to be globally abundant in anaerobic habitats and important for anaerobic hydrocarbon degradation^10,11^. In their article, Katayama et al.^9^ describe how they obtained the first axenic culture from this phylum. Again, patience was key, as this required a 3-year enrichment from aquatic samples in the vicinity of natural-gas deposits. Physiological characterization points to a metabolism based on sugar degradation coupled to hydrogen production, as earlier postulated by omics techniques^10,11^. Interestingly, the strain cannot tolerate high levels of hydrogen and thus grows better in co-culture with a hydrogen-consuming methanogenic archaeon, suggesting a syntrophic lifestyle. It is therefore tempting to speculate that at least this *Atribacter* species thrives in anoxic ecosystems thanks to its ability to act as a syntrophic partner of hydrogen-scavenging organisms such as methanogens. If confirmed by further ecological studies, this syntrophy could play a role in the methane cycle, which is fundamentally linked with global warming. Thus, understanding these processes in greater detail may be important for future predictions and model development of climate change.

## Bacteria with a membrane-enclosed nucleus?

Although previous omics approaches provided a good hint towards the ecological role of *Atribacter*, they fell short in predicting the organism’s fascinating cell biology. Having obtained an axenic culture, Katayama et al.^9^ were able to shed light on the cell biology of the first representative of the OP9 candidate phylum, now re-named as *Atribacterota*. They used diffraction-limited light microscopy, scanning electron microscopy, transmission electron microscopy (TEM) of thin sections, and cryo-electron tomography to reveal an unusual cell plan with three layers resembling lipid membranes.

**Fig. 1:**
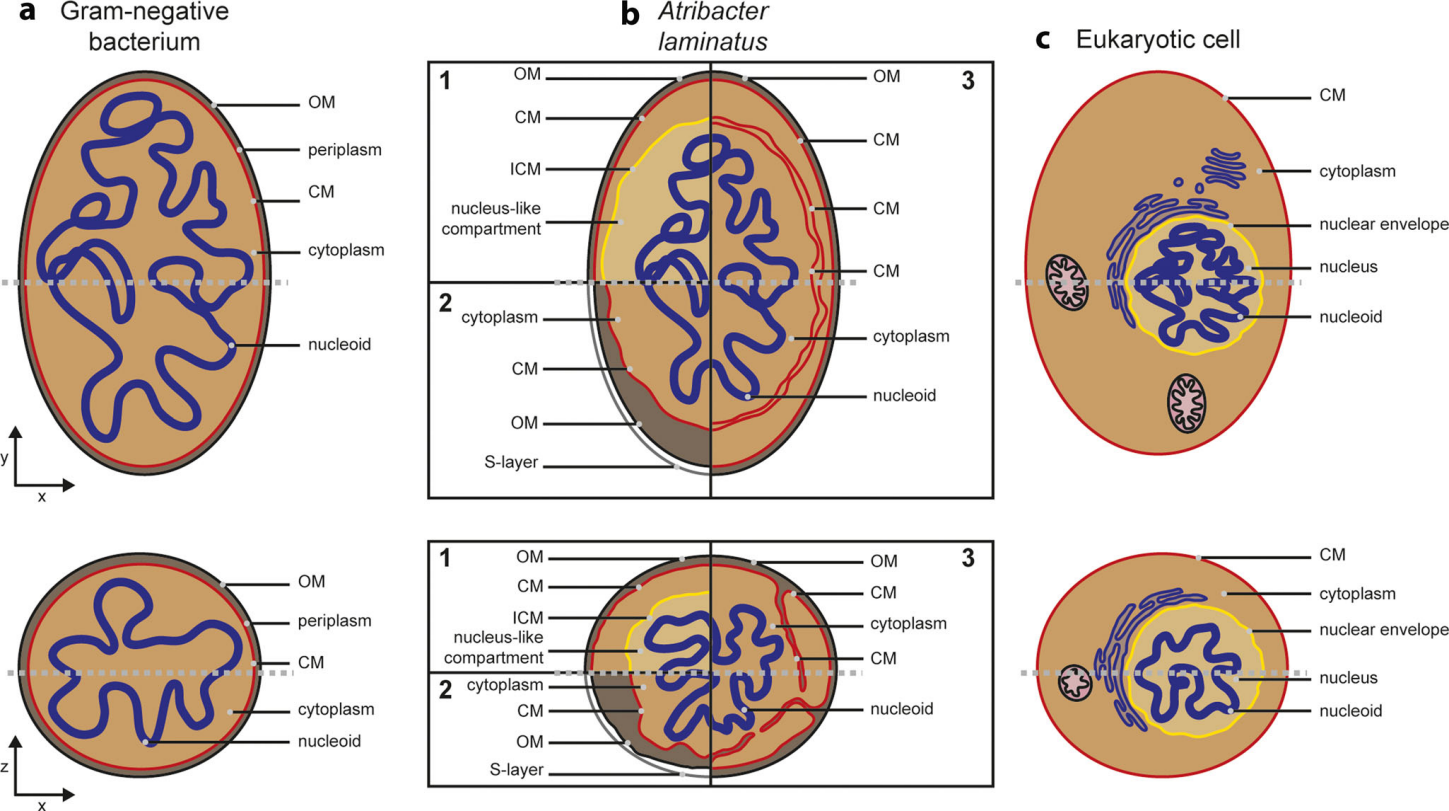
Alternative interpretations of the cell biology of *Atribacter laminatus*. Comparison of the cell plans of a typical Gram-negative bacterium (**a**), *Atribacter laminatus* (**b**), and a typical eukaryotic cell (**c**), displayed both in the xy-plane (top row) and the xz-plane (bottom row). The gray dashed lines indicate the plane illustrated in the other orientation. Shown are three possible interpretations of the cell plan of *A. laminatus*. In scenario 1, favored by Katayama et al.^9^, *A. laminatus* has a nucleus-like compartment. Scenario 2 is reminiscent of a typical Gram-negative with an additional S-layer as outer layer. In scenario 3, the cytoplasmic membrane shows extensive invaginations. OM outer membrane, CM cytoplasmic membrane, ICM intra-cytoplasmic membrane.

Katayama et al.^9^ interpret the cell plan of *A. laminatus* in terms of a diderm, Gram-negative cell envelope (with an outer membrane (OM) and a cytoplasmic membrane (CM)) and an additional intracytoplasmic membrane (ICM). This additional membrane would enclose a compartment that takes up a large part of the cell’s volume and includes the nucleoid (scenario 1 in Fig. 1). As an alternative interpretation, the authors mention that *A. laminatus*’s cell plan could consist of (from inside to outside): a cytoplasm enclosed by the CM, an enlarged periplasm bordered by the OM, and an additional proteinaceous layer such as an S-layer (scenario 2 in Fig. 1).

If *A. laminatus* contains three lipid membranes as proposed by Katayama et al.^9^, this would imply that this organism has a higher amount of membrane material than other bacteria. The authors find support for this interpretation in the discovery that the genome encodes an unusually high number of proteins with trans-membrane helices, some of which are very highly expressed. In addition, several membrane proteins (including one of the two copies of the main orchestrator of cell division, FtsZ) contain N-terminal extensions, and Sec-secreted proteins appear to have unique signal peptides.

The most intriguing observation made by the authors is that both TEM of thin sections as well as staining of nucleic acids visualized by confocal microscopy indicate that the chromosomal DNA (as well as RNA) is enclosed by the (potential) intracytoplasmic membrane, a situation that would resemble the eukaryotic nucleus. Interestingly and in stark contrast to the eukaryotic nucleus, the cryo-electron tomography data shows ribosome-like structures in this potential nucleus-like compartment.

Such interpretation of the cell plan of *A. laminatus* causes a déjà-vu, as similar observations previously led to the proposal of a nucleus-like structure for bacteria within the *Planctomycetes* (for review see Wiegand et al.^12^). Although Katayama et al.^9^ present stronger data supporting their hypothesis (when compared to previous observations leading to the planctomycetal controversy), we would still like to urge for caution in interpreting *A. laminatus* as the first prokaryote with a nucleus-like compartment. The investigations on the planctomycetal cell plan inspire both an alternative interpretation and strong experimental approaches that could help determine the nature of *Atribacter*’s exceptional cell biology. First of all, it will be of key importance to identify which of the membrane-like layers is the OM, for instance by localizing OM-specific macromolecules such as porins or LPS. Another option would be to investigate where the peptidoglycan cell wall is located, for instance using fluorescently labeled precursors that get incorporated in the peptidoglycan layer, as the OM will be located on top of this. In addition, super-resolution light microscopy may be employed to determine which of the membranes is able to support a proton gradient and coupled ATP synthesis, a typical feature of (intra)cytoplasmic membranes. The recent reinterpretation of the planctomycetal cell plan has been possible only by the use of those approaches^13–16^.

Again, the literature on *Planctomycetes* (for review see Wiegand et al.^12^) inspires a third possible interpretation of the cell plan of *A. laminatus* (scenario 3 in Fig. 1): it is possible that the layers proposed by Katayama et al.^9^ as intracytoplasmic and cytoplasmic membranes are in fact one continuous structure. Large invaginations of the CM could explain the observations, as previously shown for multiple planctomycetes (for review see Wiegand et al.^12^). To verify or falsify this interpretation, it would be of key importance to analyze many cells. The planctomycetal experience shows that one can easily find a few cells that look like they comprise a nucleus-like structure, whereas one comes to a different interpretation through the analysis of many cells. We are therefore looking forward to further in-depth experiments on *A. laminatus* and hopefully on relatives that will be added to the list of cultivated organisms, from which more clarity will emerge on how to interpret this fascinating cell plan described by Katayama and colleagues.

## Concluding remarks

The study by Katayama et al.^9^ adds to the several examples of microbes, whose cultivation and characterization have challenged existing views of how prokaryotic cells are organized^4–8^. Their work clearly illustrates how important it is to pursue the tedious task of bringing species of previously elusive phyla in culture. Without doubt, further unseen bacterial cell biology awaits discovery. As the low-hanging fruits in terms of fast and easy growing microbes have been harvested, future attempts may need to focus on slower-growing organisms and organisms with complex growth requirements such as chemically complex media, gradient parameters, or the need for co-cultivation. Future funding schemes need to acknowledge these challenges, as typical funding periods may be too short to support such discoveries. However, unearthing the microbial diversity not only reveals fascinating new biology, but also contributes to our understanding of global fluxes of matter and climate change, and enables future biotechnological applications.
